# The role of plants and soil properties in the enzyme activities of substrates on hard coal mine spoil heaps

**DOI:** 10.1038/s41598-021-84673-0

**Published:** 2021-03-04

**Authors:** Agnieszka Kompała-Bąba, Wojciech Bierza, Edyta Sierka, Agnieszka Błońska, Lynn Besenyei, Gabriela Woźniak

**Affiliations:** 1 Faculty of Natural Sciences, Institute of Biology, Biotechnology and Environmental Protection, University of Silesia in Katowice, Jagiellońska 28, 40-032 Katowice, Poland; 2 School of Sciences, Faculty of Science & Engineering, University of Wolverhampton, Wulfruna Street, Wolverhampton, WV1 1LY UK

**Keywords:** Ecology, Environmental sciences, Urban ecology

## Abstract

Knowledge about biotic (plant species diversity, biomass) and/or abiotic (physicochemical substrate parameters) factors that determine enzyme activity and functional diversity of the substrate on hard coal spoil heaps is limited. Spontaneously developed vegetation patches dominated by herbaceous species commonly occurring on these spoil heaps: grasses (*Poa compressa, Calamagrostis epigejos*) and forbs (*Daucus carota*, *Tussilago farfara*), were examined. The activity of dehydrogenase and alkaline phosphatase was twice as high in plots dominated by grass species compared with those dominated by forbs. Significant positive correlations were found between the activity of dehydrogenase and alkaline phosphatase with pH, available P, soil moisture, and water holding capacity and negative correlations between the activity of urease and soil organic carbon. Strong positive correlations were found between values for Shannon–Wiener diversity index, evenness, species richness and soil functional diversity in plots dominated by grasses. We found that the soil physicochemical parameters had a greater impact on enzyme activity of the substrate than plant biomass and species diversity. However, grasses, through their extensive root system, more effectively increased enzyme activity and health of the substrate than other herbaceous species, and as they stabilize the substrate and form dense plant cover, they can be recommended for reclamation purposes.

## Introduction

Soil enzymes play a crucial role in soil functioning particularly in the cycling of carbon contained within dead organic matter, acquisition of nutrients from organic resources and decomposition of xenobiotics^1^. They are also associated with proliferating soil microbial communities, which play a key role in many soil processes and the delivery of essential soil ecosystem services^2^.

Enzyme activity in the soil depends on many factors such as: physicochemical properties of soil (soil reaction, content of soil organic matter, total nitrogen, phosphorus and sulphur as well as heavy metal pollution), climatic conditions and cropping systems^3–7^. Moreover, soil enzyme activity also depends on the abundance and diversity of microbial communities^8^. As already stated the activity of dehydrogenase has a close relationship with the populations of soil microbes and the diversity of the microbial community structure^9^. Also phosphatase and urease activity are closely related to the populations of soil microbes^10,11^. Many authors indicate that the composition of microorganisms in the soil can be changed depending on the diversity of the plant community (particularly the occurrence of dominant species) growing on it and the extent of the vegetation period^12,13^. Differences in the microbial community, thus soil enzyme activity, may also result from differences in the number of plant's small roots and their metabolic activity, and the amount and chemical composition of litter produced by a specific plant^14^. The coexistence of multiple plant species and their diversity can increase the complexity of soil microbial communities and stimulate soil enzyme activity by increasing the heterogeneity of organic compounds found in the soil during litter decomposition and root activity^15,16^. The close relationship of plant community composition, including species richness and functional group assembly, influences soil functions such as soil microbial biomass and activity has been conducted by many researchers^17–19^. Therefore, the presence of plants seems to have a key impact on the enzymatic activity of the soil, because the chemical composition of plant residues and thus the soil nutrient status affect microbial activity and microbial community structure^20^. For all of these reasons soil enzymes are used as an index of soil microbial activity and can react quickly to changes in environmental conditions^21–23^, microbial community structure^24^ and vegetation diversity^25,26^. A more accurate understanding of biochemical changes occurring in soil can be achieved using a biochemical soil fertility index (M_w_) that includes dehydrogenase, urease, acid and alkaline phosphatase activities, as well as soil organic carbon content^27^. Until now many studies have been conducted on the activity of soil enzymes in agricultural areas^28^, forest ecosystems, as well as on areas contaminated with heavy metals, open cast lignite mining or spoil heaps after brown coal mining^1,3,6,29,30^. However, only a few studies dealing with the activity of soil enzymes have been carried out on spoil heaps after hard coal mining^31^. Coal mine spoil heaps provide a good example of newly established habitats that differ from the natural ecosystems present in the surrounding landscape^32^. Many studies have revealed that these sites have been colonized by living organisms through spontaneous succession, providing novel species compositions of flora and fauna, where frequently one species dominates the floristic composition^33^. Such ecosystems cannot be returned to the historical state however, further biotic and abiotic shifts are possible that need comprehensive studies. The relationships between plants and the soil biota can change during succession taking place on a specific type of post-industrial site. In the early stages of succession soil organisms may depend on the presence of a specific combination of plant species that are responsible for organic soil layer formation^34,35^. These kinds of post-industrial sites provide an opportunity to study the relationships between the dominant plant species, along with their species richness, diversity, and biomass, together with abiotic substrate parameters and soil enzyme activity.

The aim of the study was to determine which group of factors: abiotic (physicochemical substrate parameters) and biotic (plant species diversity, biomass of a dominant plant species, functional groups of species) determine the enzyme activity of substrates of hard coal spoil heaps. Moreover, we aimed to examine the relationship between vegetation diversity and substrate functional diversity. We hypothesized that on coal mine spoil heaps vegetation cover (or chosen functional groups of species) will have a greater influence on enzyme activity and functional diversity of the substrate than its physicochemical parameters.

## Materials and methods

### Site description

**Figure 1:**
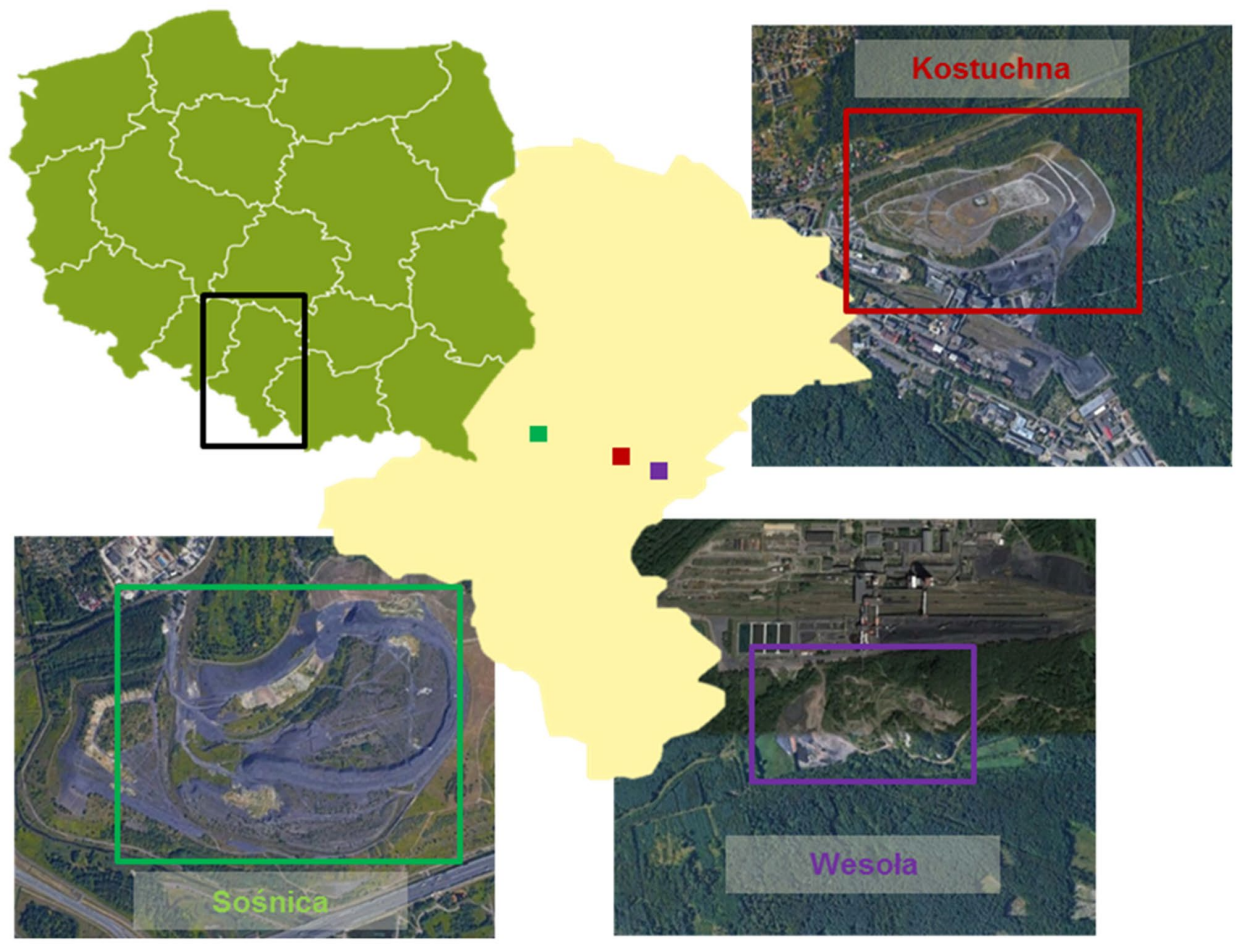
Location of the investigated coal mine spoil heaps in the Silesian Uplands (southern Poland) with a satellite image of each one. Satellite images were screen shots from open source Google maps (https://www.google.com/maps/).

The study was carried out on hard coal mine spoil heaps located in the Silesian Uplands (Southern Poland). We investigated three spoil heaps which were not technically reclaimed: “Sośnica” in Gliwice (50° 16′ 22′′ N, 18° 44′ 43′′ E), “Wesoła” in Mysłowice (50° 10′ 28′′ N, 19° 5′ 44′′ E) and “Kostuchna” in Katowice (50° 11′ 4′′ N, 19° 0′ 33′′ E) (Fig. 1). Spontaneous succession on the investigated areas began about 25 years ago. The studied heaps are generally irregular in shape, and built of carboniferous gangue with unfavourable soil texture (mainly claystone and siltstone, also sandstone, conglomerate, coal shale) with admixtures of coal^36,37^. Such sites are often subject to extreme abiotic conditions e.g. low water retention, lack of water, fast drying of the surface layer, low nutrient availability, low levels of organic matter, high temperature (reaching endogenous thermal activity) and different salinity levels (crystallized salt can sometimes be observed)^38^. Despite their anthropogenic origin, the substrate of coal mine spol heaps does not contain high concentrations of heavy metals and other dioxins compared to other post-industrial sites (e.g. lead and zinc spoil heaps)^39,40^.

### Vegetation, biomass and soil sampling

For this research a total of 75 sample plots in the shape of a circle (6 m in diameter) were established on the spoil heaps in July 2016 to examine the relationships between physicochemical properties of the substrate, the vegetation dominated by chosen plant species, commonly occurring on these spoil heaps (Table 1) (15 plots for each dominant species), and the enzyme activity of the substrate. Moreover 15 control plots without vegetation were selected to examine if plants have any influence on enzyme activity of the spoil heap substrate. Plots were located at least 10 m from each other. All the study plots were established on flat terrain (on the tops of spoil heaps) which had been leveled by machines during their construction, to avoid differences between them regarding exposure to sunlight and susceptibility to erosion. In addition, unevenness of the terrain surface, such as micro-depressions and micro-elevations were avoided, as they may have affected the accumulation of organic matter, or water retention, and thus obscured the effects of the plant species. Two herbaceous groups were chosen for detailed investigation: grass species (monocotyledons) (*Calamagrostis epigejos, Poa compressa*) and forb species (dicotyledons) (*Daucus carota, Tussilago farfara*) (Table 1)*.* In each study plot, the percentage cover of vascular plant species (estimated as cover for each species), was estimated according to the following scale: 1, 2, 5, 10% and then at 10% intervals up to 100%^41^. The above-ground part of the plant biomass was collected from 0.25 m^2^ of the quadrats. In each plot the rhizosphere substrate (in the case of vegetated plots) and bulk substrate were taken from three points at a depth of 10 cm. Substrate samples for enzyme analysis were placed into plastic bags and immediately transported to the laboratory, where the samples were sieved through a 2 mm mesh sieve. The prepared material was stored in polyurethane bags at 4 °C until analysis. In the laboratory, plant biomass samples were first dried at 105 °C for 24–48 h in a laboratory oven and then weighed to determine their dry biomass^42^.

**Table 1:** Characteristics of plots dominated by selected plant species: monocotyledons (grasses*) and dicotyledons (forbs**).

Dominant species	life form, life span; ecological group; life strategy, height; family, abundance	Characteristics of the vegetation and species composition
Daucus carota**	Hemicryptophyte /biennial; meadow species CR, 40–100 cm, *Asteraceae,* abundance in patches 5–30%	Species richness 12.8; total cover of vegetation 54%; species composition (*Calamagrostis epigejos, Lotus corniculatus, Hieracium piloselloides, Picris hieraciodes, Matricaria martima* subsp. *inodora*)
Tussilago farfara**	Geophyte/perennial; ruderal species; CSR; < 30 cm, *Asteraceae,* abundance in patches 20–40%	Species richness 8.3; total cover of vegetation 46%; species composition (*Calamagrostis epigejos, Daucus carota, Chamaenerion palustre, Hieracium piloselloides*)
Poa compressa*	Hemicryptophyte; perennial species, xerothermic species; CSR, 20–80 cm, *Poaceae,* abundance in patches 20–40%	Species richness 13.9, total cover of vegetation 70%; species composition (*Leontodon autumnalis, Lotus corniculatus, Daucus carota, Plantago lanceolata, Achillea millefolium*; *Medicago lupulina, Echium vulgare Picris hieracioides*, *Calamagrostis epigejos*)
Calamagrostis epigejos*	Geophyte/perennial; ruderal species; C, 1.45 cm, *Poaceae,* abundance in patches 10–80%	Species richness 12.8, total cover of vegetation 73%, species composition (*Picris hieracioides, Oenothera* sp., *Senecio viscosus*, *Solidago gigantea*, *Poa compressa*)

### Calculation of species diversity indices

Shannon–Wiener diversity index (H’) (Eq. (1)), Evenness (E) (Eq. (2)) and dominance index (1-D) (Eq. (3)) were calculated for sample plots covered by vegetation^43^. The Shannon–Wiener diversity index assumes that individuals are randomly sampled from an independent large population, and all the species are represented in the sample. It is calculated according to the formula:

1 $${H}{^{\prime}}=\sum_{i=1}^{s}pi\,\text{ln}\,pi,$$

where *S* is the number of species, *pi* is the proportion of the individual species cover relative to the total cover.

Evenness (E) describes the way the species are distributed in the community. It has a value between 0 and 1 (complete evenness). It is calculated according to the formula:

2 $$E= H{^{\prime}}/{H}{^{\prime}}\text{max}= H{^{\prime}}/lnS,$$

where *H*′ = observed diversity; *H*′*max* = maximum diversity for a given number of species (S).

Dominance index (1-D) ranges between 0 and 1. The greater the value of D, the greater the sample diversity. It is calculated according to the formula:

3 $$D= \sum_{i=1}^{s}pi.$$

### Enzyme assays

The activities of dehydrogenase, acid and alkaline phosphatase as well as urease were chosen due to their widespread occurrence and their significant role in the transformation of organic matter^44^. Dehydrogenase activity is an indicator of the oxidation and health status of biological systems and is a marker of microbial activity and the intensity of microbial metabolism in the soil since it only occurs in living microorganisms^45^. Phosphatases play a crucial role in the phosphorus cycle and decomposition of organic phosphorus in various terrestrial ecosystems^46^. Acid phosphatase (extracellular) and alkaline phosphatase (intracellular) are the major enzymes responsible for the mineralization of organic phosphorus^47^. Urease, that provides nitrogen to plants is an extracellular enzyme produced by microorganisms and is involved in the decomposition of urea into ammonium carbonate, making it a key enzyme in the nitrogen cycle^48^.

Dehydrogenase (EC 1.1) activity was determined by reduction of 2,3,5-triphenyltetrazolium chloride (TTC) to triphenylformazan (TPF) by the method developed by Schinner et al.^49^. Urease (EC 3.5.1.5) activity was determined according to the Alef and Nannipieri^50^ protocol based on the incubation of substrate samples with urea solution. The assays of acid phosphatase (EC 3.1.3.2) and alkaline phosphatase (EC 3.1.3.1) activity were determined by measuring the p-nitrophenol (PNP) released by phosphatase activity after soil incubation with buffered (pH 6.0 for acid phosphatase and pH 11.0 for alkaline phosphatase) sodium p-nitrophenyl phosphate (115 mM) solution^49^.

On the basis of enzymatic activity and soil organic carbon (SOC) content, a potential biochemical soil fertility index (M_w_) (Eq. (4)) was computed from the formula proposed by Wyszkowska and Wyszkowski^51^:

4 $${\text{M}}_{{\text{w}}} = \, \left( {{\text{URE 1}}0^{{ - {1}}} + {\text{DAH }} + {\text{ ACP}} + {\text{ALP}}} \right) \, \% {\text{SOC}},$$

where: URE—is urease, DAH—dehydrogenase, ACP—acid phosphatase, ALP—alkaline phosphatase, and SOC—soil organic carbon.

### Soil functional diversity

Soil functional diversity measures the actual functioning of the whole microbial community in contrast to microbial community metabolisms and microbial diversity as determined by culture-based physiological profiling^52^.

To calculate soil functional diversity we applied the formula used by Rodriguez-Loinaz et al.^53^. From the values of all measured enzyme activities, soil functional diversity was determined using the Shannon-Weiner diversity index according to the formula:

$$Soil \,{H}{^{\prime}}=\sum_{i=1}^{s}pi\,\text{ln}\,pi,$$

where *pi* is the ratio of the activity of a particular enzyme to the sum of the activities of all enzymes.

The order of magnitude of the values obtained for the different enzyme activities can vary considerably depending on the specific activity being determined, thus leading to some enzyme activities having more weight than others during the calculation of the diversity index. To give all enzyme activities the same weight/relevance, the value obtained for each enzyme activity was divided by the highest value found for that specific activity in the whole set of samples, and then multiplied by 100. Thus for each enzyme activity, the percentage of the maximum value found for that specific activity in the whole set of samples was calculated.

### Substrate physicochemical analyses

Substrates for the physicochemical analyses were air-dried in the laboratory to constant weight at room temperature and sieved (through 2 mm or 0.25 mm mesh depending on the analysis). Substrate pH, in 1 M KCl (potential acidity) and in water suspension (pH in H_2_O) (actual acidity) and electrical conductivity (EC) (substrate to solution ratio 1:2.5), were measured after 24 h of equilibration^54^. The measurements were performed by the potentiometric method using a SEN 81st TI X electrode. Substrate organic carbon content (SOC) was determined by the Tiurin method modified by Simakov^54^. Total N (TN) was determined by the Kjeldahl method^54^. Content of available forms of phosphorus (P_2_O_5_) was estimated according to the Polish Norm PN-R-04023:1996 based on the Egner-Riehm method. The concentration of available Mg was measured by spectrometric analysis^54^. Moisture was determined after drying overnight at 105 °C. Water holding capacity (WHC%) was measured by the gravimetric method^55,56^. Water was added to substrate until the soil was saturated. The substrate and water were then placed in a plastic tube with a 1.5 cm diameter hole in the bottom covered by two layers of 2 mm mesh. The top of the container was sealed with plastic to reduce evaporation from the surface, and the substrate was allowed to drain overnight. The moisture content of the soil on a dry weight basis at this point was termed the water holding capacity (WHC) of the soil^55,56^.

### Statistical analysis

Welch’s ANOVA and Tukey’s post hoc test were used to test the differences in physicochemical substrate parameters, and soil enzyme activities between plots covered by the selected dominant plant species. The normality of the variables was examined with the Shapiro–Wilk test after a Box-Cox transformation. The relationships between soil enzyme activities, plant biomass of the dominant species as well as species richness and species diversity indices (Shannon–Wiener diversity index (*H*′), Evenness (*E*) and dominance index (1-*D*) of the studied plots were evaluated using Pearson’s correlation coefficient. Diversity indices (Shannon–Wiener diversity index (H’), Evenness (E) and dominance index (1-D)) were calculated for sample plots covered by vegetation (in our studies dominated by grasses and other forbs) in JUICE ver. 7.1^43^.

In order to show relationships between chosen physicochemical substrate parameters, species diversity and biomass, and the activity of investigated soil enzymes RDA was applied using Canoco 5.0 for Windows^57^ and forward selection was used to choose those variables that compared community above-ground biomass with soil chemical properties and soil enzyme activities. The varpart function in the vegan package in R project^58^ was used to summarize the variation in soil enzyme activity using physicochemical substrate parameters, species diversity and biomass. Pearson’s correlation coefficient was used to show the relationship between plant species diversity calculated for both the whole vegetation and separately for grasses or forbs, and substrate functional diversity. Statistical analyses were conducted using Statistica v.13.1^59^.

## Results

### Substrate physicochemical parameters

The properties of the substrate in the plots covered by the studied vegetation types (with dominant plant species) differed significantly for substrate reaction (pH in H_2_O and KCl) and available P (except for those plots with *C. epigejos*) from plots without vegetation (control). In contrast no significant differences were found between both types of plots with reference to content of total N (TN), EC and moisture. The highest soil organic content (SOC) content was found in plots dominated by *T. farfara*, and the highest WHC was measured in plots dominated by *P. compressa* (Table 2).

**Table 2:** Substrate parameters of different vegetation plot types (mean ± SE). No statistical differences are marked by the same letter (p < 0.05).

Parameters	*C. epigejos* (n = 15)	*D. carota* (n = 15)	*P. compressa* (n = 15)	*T. farfara* (n = 15)	Control (n = 15)
pH H_2_O	6.13 ± 0.25ab	6.44 ± 0.14ab	6.88 ± 0.15a	5.62 ± 0.35b	4.35 ± 0.24c
pH KCl	5.46 ± 0.28ab	5.95 ± 0.19ab	6.26 ± 0.16a	5.15 ± 0.38b	3.90 ± 0.27c
EC (mS cm^−1^)	0.24 ± 0.03a	0.35 ± 0.05a	0.52 ± 0.13a	0.28 ± 0.04a	0.26 ± 0.08a
TN (%)	0.26 ± 0.04a	0.26 ± 0.02a	0.31 ± 0.04a	0.27 ± 0.03a	0.26 ± 0.02a
SOC (%)	10.29 ± 1.72b	12.43 ± 1.15ab	13.87 ± 1.81ab	18.02 ± 2.39a	12.14 ± 1.36ab
available Mg (mg kg^−1^)	291.80 ± 21.01ab	322.37 ± 10.66a	306.13 ± 17.03ab	191.47 ± 20.79b	229.20 ± 32.06b
available P (mg P_2_O_5_ kg^−1^)	9.66 ± 2.19ab	10.03 ± 1.52a	10.41 ± 1.65a	12.03 ± 2.14a	4.30 ± 0.83b
Moisture (%)	2.45 ± 0.16a	2.51 ± 0.28a	2.57 ± 0.25a	2.24 ± .020a	2.12 ± 0.16a
WHC (%)	29.01 ± 1.04ab	26.12 ± 1.17bc	31.62 ± 1.49a	24.24 ± 1.32bc	23.73 ± 1.39c

### Plant diversity and biomass of the dominant species

Plots dominated by *T. farfara* differed significantly from other plots with reference to species richness and diversity measured by the Shannon–Wiener index as well as the dominance index (Tables 1, 3). Mean values of Shannon–Wiener and dominance index from *T. farfara* plots were the lowest in comparison to plots dominated by other plant species. In contrast, the highest values for the Shannon–Wiener diversity index, as well species richness, were recorded in the plots dominated by *P. compressa* and *D. carota*. These plots were also characterized by the lowest values for Evenness. The highest biomass was recorded in the plots of *C. epigejos* and *T. farfara* compared with those of *P. compressa* and *D. carota*.

**Table 3:** Comparison of plots dominated by grasses (*C. epigejos* and *P. compressa*) and forb species (*D. carota* and *T. farfara*) with reference to Shannon–Wiener diversity, Evenness and biomass of the dominant species (mean ± SE).

	*C. epigejos* (n = 15)	*P. compressa* (n = 15)	*D. carota* (n = 15)	*T. farfara* (n = 15)
Shannon–Wiener index (*H’*)	1.58 ± 0.13a	1.92 ± 0.06a	1.82 ± 0.09a	1.11 ± 0.11b
Species richness (*No*)	12.80 ± 1.16ab	13.87 ± 0.90a	12.80 ± 0.86ab	10.53 ± 2.58b
Evenness (*E*)	0.62 ± 0.04bc	0.74 ± 0.01a	0.72 ± 0.02ab	0.53 ± 0.03c
Dominance index (1-*D*)	0.38 ± 0.04b	0.25 ± 0.01c	0.29 ± 0.03bc	0.53 ± 0.04a
Biomass	52.81 ± 9.66a	11.66 ± 1.74b	8.78 ± 1.78b	38.33 ± 6.20a

No statistical differences are marked by the same letter (p < 0.05).

### Substrate enzyme activity and substrate functional diversity

**Figure 2:**
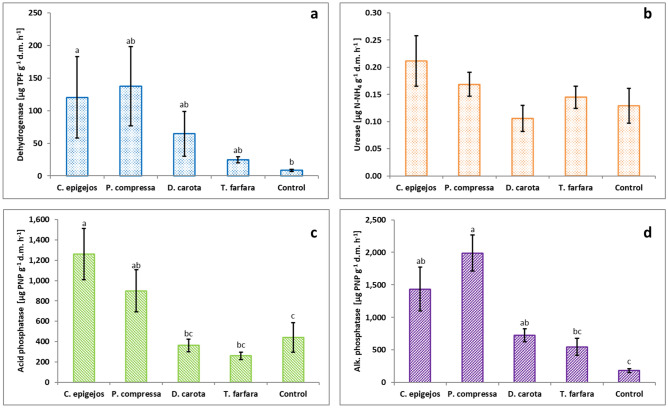
Activity of soil enzymes in plots dominated by selected plant species and control plots. Data are the means ± SE (n = 15). No statistical differences are marked by the same letter (*p* < 0.05). *d.m.* dry mass of soil.

**Figure 3:**
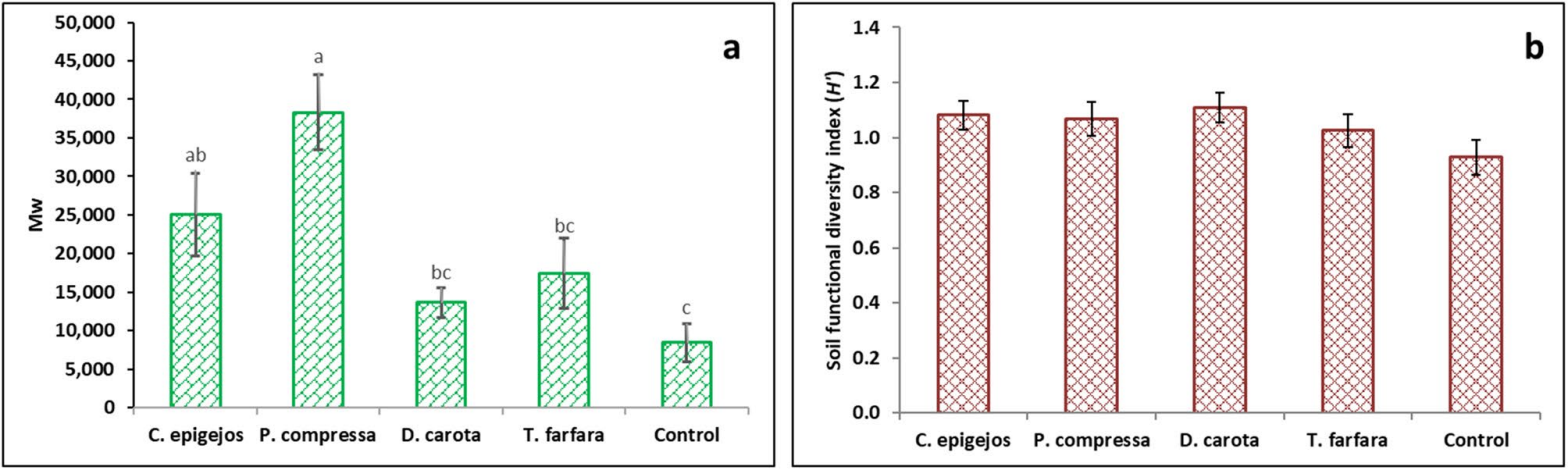
Potential biochemical soil fertility index (M_w_) (**a**) and substrate functional diversity index (**b**) in plots dominated by selected plant species and the control plots. Data are the means ± SE (n = 15). No statistical differences are marked by the same letter (*p* < 0.05).

The activity of the soil enzymes and M_w_ was highest in plots dominated by grass species compared with plots of forb species. This was especially evident in the case of dehydrogenase and alkaline phosphatase (Fig. 2) as well as M_w_ (Fig. 3a). Urease activity in substrate from plots dominated by grasses was not significantly different to the activity of this enzyme in plots dominated by forb species and control plots (Fig. 2b). Alkaline phosphatase activity was significantly higher in substrates under *P. compressa* compared with plots dominated by the other species. Plots dominated by *T. farfara* were characterized by low enzyme activity of the substrate, which was similar to plots without vegetation. Also in the plots dominated by *D. carota* no high enzyme activity in the substrate was recorded, only in the case of alkaline phosphatase was the activity of this enzyme significantly higher than in the control plots. There was no statistically significant difference in soil functional diversity between the investigated plot types (Fig. 3b).

### The influence of physicochemical parameters on the enzyme activity of the substrate

Significant positive correlations were found between substrate pH (in H_2_O) and dehydrogenase, alkaline phosphatase and substrate functional diversity. Similarly, significant positive correlations were found between dehydrogenase, alkaline phosphatase, substrate functional diversity and available P in the substrate. Dehydrogenase activity was also positively correlated with available Mg. Moreover, M_w_ was positively correlated with substrate pH (in H_2_O and 1M KCl), TN and SOC content. A strong positive correlation was found between dehydrogenase, both alkaline and acid phosphatase activity and substrate water holding capacity (WHC). Positive correlations were also found between dehydrogenase and alkaline phosphatase and soil moisture. Negative correlations were found between urease activity, acid phosphatase and SOC. No significant correlations were found between soil enzyme activities and total N (Table 4).

**Table 4:** Pearson’s correlation coefficient values between chosen physicochemical parameters, soil enzyme activity, functional diversity of the substrate and potential biochemical soil fertility index (M_w_).

	Soil H’	Dehydrogenase	Urease	Acid phosphatase	Alkaline phosphatase	M_w_ index
pH_H2O_	0.251*	0.247*	0.128	0.084	0.660*	0.463*
pH_KCl_	0.224	0.157	0.076	0.034	0.606*	0.448*
EC (mS cm^−1^)	0.050	− 0.089	− 0.166	− 0.117	0.171	0.171
TN (%)	0.034	− 0.084	− 0.154	− 0.075	0.108	0.482*
SOC (%)	0.028	− 0.150	− 0.251*	− 0.199	− 0.022	0.447*
Available Mg (mg kg^−1^)	0.071	0.220	− -0.074	− 0.089	0.038	− 0.017
Available P (mg kg^−1^ )	0.348*	0.489*	0.113	0.093	0.273*	0.054
Moisture (%)	0.146	0.446*	0.123	0.129	0.254*	− 0.099
WHC (%)	0.201	0.452*	0.208	0.260*	0.555*	0.210

Significant correlations (*p* ≤ 0.05) are marked with *.

### The influence of plant diversity and plant biomass on enzyme activity of the substrate

Pearson’s correlation coefficients were calculated for soil enzyme activities, plant diversity indices and M_w_ index for the whole vegetation and separately for groups of plots dominated by grass and forb species (Table 5). No significant correlations were found between urease activity and most diversity indices calculated for grasses, forbs and the whole vegetation. Urease was only negatively correlated with Evenness in the case of grass species. In contrast, strong correlations (**p ≤ 0.01 or *p ≤ 0.05) were found between alkaline phosphatase and almost all diversity indices calculated for grass species, forbs and total number of species. Strong correlations (**p ≤ 0.01) were found between acid phosphatase and Shannon-Wiener diversity and Evenness calculated for grasses as well as forbs. We did not find significant correlations between soil functional diversity (soil *H*′) and plant diversity for the whole vegetation in the data set or between soil functional diversity and biomass. Strong correlations were found only in grass dominated sample plots, with Shannon-Wiener diversity (*H*′), Evenness, species richness, dominance and soil functional diversity, and for plots dominated by forb species with Shannon–Wiener diversity, species richness and soil functional diversity. M_w_ index was positively correlated with almost all plant diversity indices calculated for grasses and whole vegetation.

**Table 5:** Pearson’s correlation coefficients between enzyme activity, potential biochemical soil fertility index (M_w_), substrate functional diversity (soil H′) and diversity indices calculated for the whole dataset and according to functional groups (e.g. grasses, forb species).

	Grasses				Forbs				Whole vegetation				Biomass
	*H'*	*No*	*E*	1*-D*	*H'*	*No*	*E*	1*-D*	*H'*	*No*	*E*	1*-D*	
Dehydrogenase	0.073	0.105	0.097	− 0.096	0.009	0.058	0.001	− 0.012	0.077	0.129	0.048	− 0.102	0.198
Urease	− 0.215	0.130	− 0.410*	0.107	0.118	0.041	0.131	− 0.095	0.029	0.084	− 0.039	− 0.003	0.167
Acid phosphatase	− 0.391*	0.005	− 0.427*	0.042	0.267*	0.170	0.278*	− 0.284*	0.150	0.001	0.003	− 0.150	0.248
Alkaline phosphatase	− 0.145	0.282*	− 0.342*	0.032	0.463**	0.363*	0.460**	− 0.474**	0.367*	0.344*	0.278*	− 0.347*	− 0.031
Soil *H'*	− 0.169	− 0.113	− 0.081	− 0.179	0.062	− 0.179	0.042	− 0.061	0.079	0.049	0.045	0.104	0.119
Soil *H'* grasses dominated plots	0.475*	0.393*	0.424*	− 0.491**	− 0.119	0.181	− 0.411*	0.283	0.048	0.211	− 0.142	− 0.071	0.122
Soil *H'* forbs dominated plots	− 0.430*	− 0.535*	− 0.189	− 0.040	− 0.070	− 0.302	− 0.040	− 0.249	0.091	− 0.042	0.179	− 0.081	0.065
M_w_ index	0.426*	0.284	0.433*	− 0.469*	0.042	0.231	0.008	− 0.036	0.323*	0.318*	0.294*	− 0.342*	0.033

*H′* Shannon–Wiener plant diversity index, *No* species richness, *E* Evenness, 1-*D* dominance index, soil *H’* soil functional diversity.

Significant correlations (*p* ≤ 0.05) are marked with * or (p ≤ 0.01) with**.

### Relationships between physicochemical parameters, plant diversity, biomass and enzyme activity

**Figure 4:**
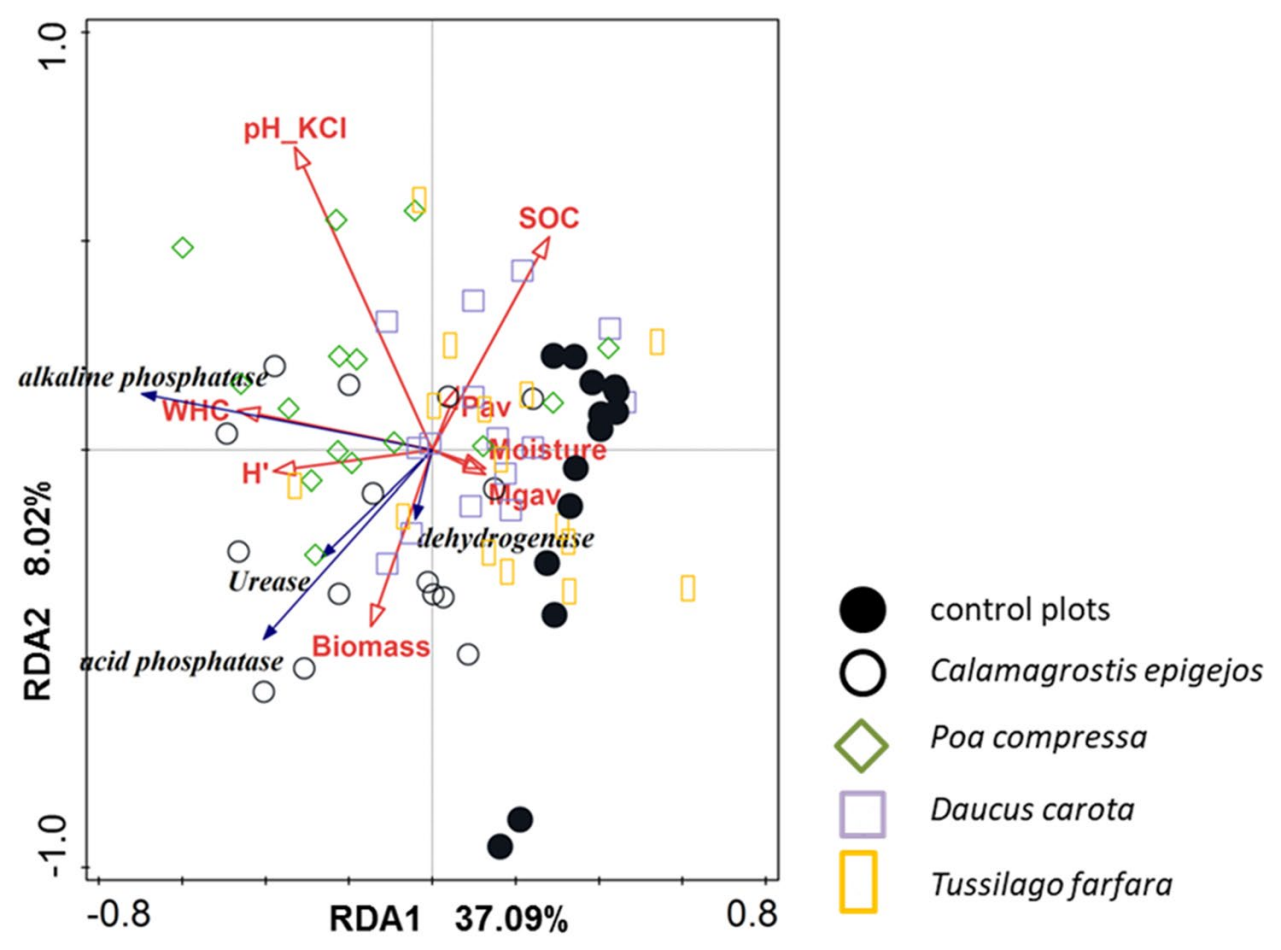
The relationships between activity of soil enzymes (blue arrows) in plots (symbols) dominated by selected grass and forb species and physicochemical substrate variables, plant diversity (H′) and vegetation biomass (both red arrows) on coal mine spoil heaps (*Mgav* available magnesium, *H*′ Shannon–Wiener diversity index, *Pav* available phosphorus, *SOC* soil organic carbon, *WHC* water holding capacity).

**Figure 5:**
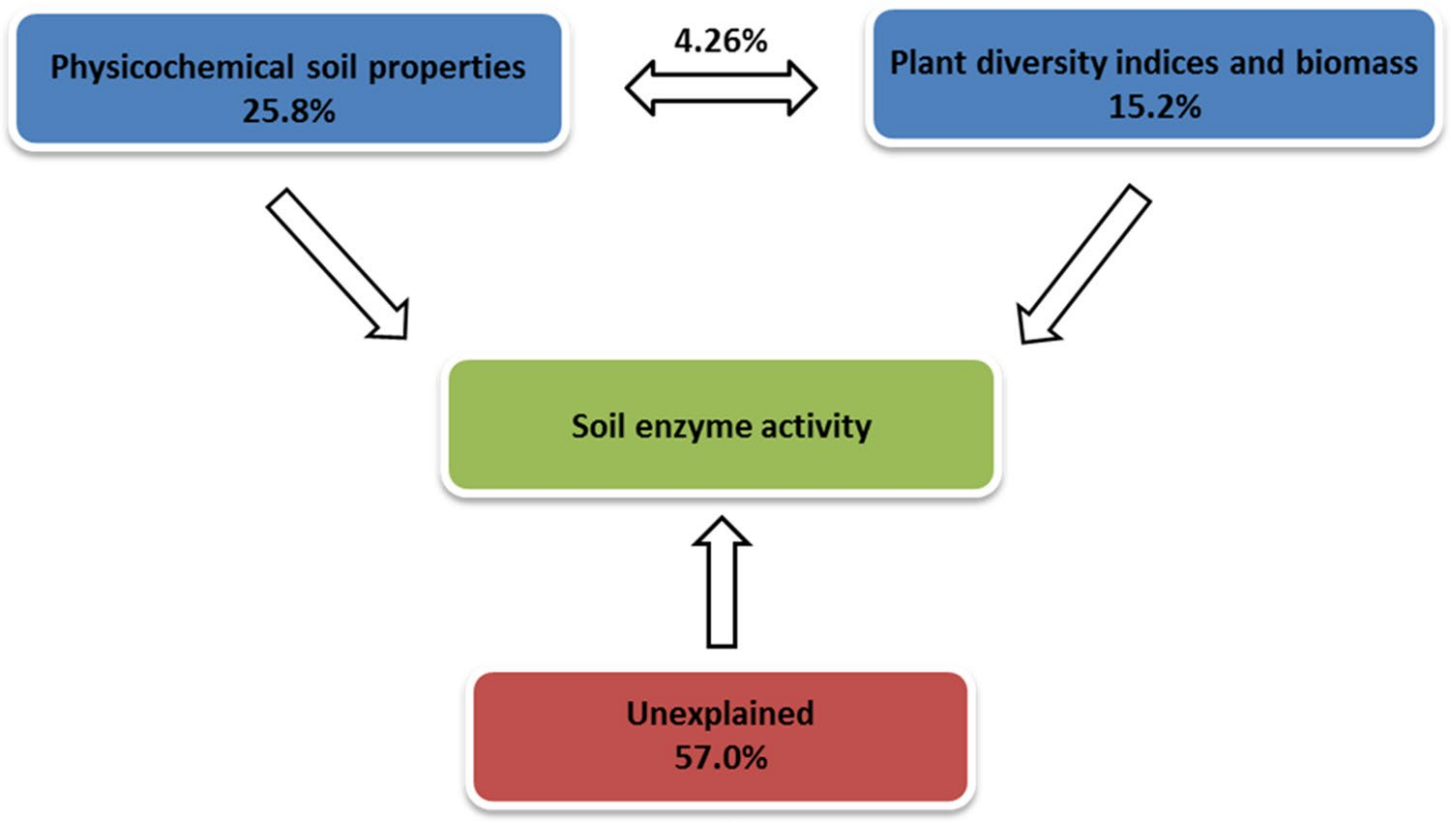
Variance partitioning analysis of soil enzyme activity explained by physicochemical soil properties, plant diversity and biomass.

RDA was conducted to identify whether physicochemical factors, plant species diversity indices, or biomass had the most important effect on enzyme activity of the substrate (Fig. 4, Table 6). All explanatory variables used in the analysis accounted for 45.5% of the total variation. Of the 14 variables taken into consideration in the analysis 8 of them (pH_KCl_, WHC, moisture, SOC, available Mg and P, H′ and biomass) made a significant contribution to explaining the total variation in the dataset (Table 6). The highest contribution (15.7%) was made by water holding capacity (WHC). Variance partitioning analysis showed that physicochemical soil parameters and plant diversity indices independently explained 25.8% and 15.2% of the total soil enzyme activity respectively. Interactions between soil properties and vegetation diversity explained 4.26% of the variance (Fig. 5).

**Table 6:** Significant physicochemical variables of the substrate and biotic parameters that explain the enzyme activity of hard coal mine spoil heaps.

Variable	Explain %	Contribution %	p
WHC (%)	8.6	15.7	0.012
pH in KCl	8.3	15.1	0.018
Mg available (mg kg^−1^)	5.8	10.6	0.010
Biomass (g)	5.3	9.7	0.022
Moisture (%)	5.1	9.3	0.032
P available (mg P_2_O_5_ kg^−1^)	4.9	9.0	0.038
SOC (%)	4.2	7.7	0.046
Shannon–Wiener diversity index H′	3.3	6.1	0.058

## Discussion

### Soil enzyme activities and physicochemical parameters

It has already been stated that enzyme activity depends on different soil properties, however, the results are sometimes ambiguous^45^. It is evident that soil enzyme activity is strongly connected with SOM in the soil, because it affects the supply of energy for microbial growth and enzyme production^60^. Most scientists have found positive correlations between enzyme activities and SOC as well as TN in human disrupted areas^29,61–63^. Baldrian et al.^1^ detected that during spontaneous succession on spoil heaps after brown coal extraction the content of SOC and TN in the topsoil layer had a significant influence on enzyme activities. Zhang et al.^64^ found that the direction of vegetation restoration had an influence on different carbon sources that significantly influenced the metabolic activity and functional diversity of the microbial community in sandy soils. In the case of our research we did not find significant correlations between SOC in the substrate and dehydrogenase activity or between SOC and activity of acid or alkaline phosphatases. In contrast to other studies, we obtained negative correlations between urease activity and SOC^53,65^. For both vegetated and unvegetated plots it is possible that most of the carbon contained in the spoil material was related to organic matter of recent or geogenic origin^66,67^. Geogenic coal is not available to microorganisms, and therefore, despite the high content of organic carbon (10–18%), the substrate in the studied plots had low available carbon sources for microorganisms^68^. The small quantity of available carbon for microorganisms in the total pool of SOC may have contributed to the lack of correlation between SOC and the activity of the studied soil enzymes, which are mainly derived from soil microorganisms^9–11^.

The results of some studies have revealed that soil pH influenced enzyme activity and soil microbial community structure^53,69^. We detected significant positive correlations between dehydrogenase and alkaline phosphatase activity as well as soil functional diversity and substrate pH. Both dehydrogenase, and alkaline phosphatase are optimum in neutral or alkaline soil pH (7.1–10)^21,45^, which is close to soil substratum pH in plots dominated by *C. epigejos*, *P. compressa* and *D. carota*. Soil pH has been reported to affect the activity of soil enzymes through different mechanisms. Changes in the ionic form of the active sites of the enzymes, which alter the three-dimensional shape of the enzymes, and affect the affinity of the substrate to the enzyme are thought to account for most of the decrease in enzyme activity observed when pH deviates from optimum^70^. Earlier studies have confirmed that the activity of these two enzymes and soil functional diversity increases with increasing soil pH^45,53,71^. However, Li et al.^29^ did not find significant correlations between pH and enzyme activities or microbial abundance with regard to reclamation treatment (control, plantation, mixed forests) on coal mine sites.

In our research we found significant positive correlations between available phosphorus and dehydrogenaseas as well as alkaline phosphatase, although available P content in the investigated substrates was relatively low, according to the Polish norm PN-R-04023 for mineral soils. Positive correlations between activity of dehydrogenase, phosphatase and available P content were also detected by Chodak and Niklińska^61^ in reclaimed heaps of lignite open-pit mines. It is known that high activity of soil phosphatases may indicate an insufficient P supply for microbes^49^. On the other hand high levels of total phosphate in soil cause a decrease of acid phosphatase activity^72^.

Water availability had a strong influence on microbial community composition as well as on soil enzyme activity because increased moisture enabled soluble organic matter to be brought into solution in the soil^45,73^. Water conditions that occur in the substrate of carboniferous gangue are generally very harsh for organisms, causing the porous surface layer of the substrate to dry-out very quickly during the summer months and the encrustation of heavily weathered slopes due to the release of large amounts of sodium^74^. In our research we found a positive correlation between soil enzyme activity and water holding capacity (WHC) as well as substrate moisture. However, the moisture within the substrate was relatively low in all plot types.

### Soil enzyme activity and vegetation

Our research confirmed the relationships between vegetation, biomass (productivity), and soil enzyme activity in the substrate of spoil heaps after hard coal mining. Such relationships had already been revealed in research conducted on semi-natural alpine meadows^26^ as well as on other post-industrial lands such as those on brown coal spoil heaps^1,23,29,34,75,76^, and surface coal mining sites^29,77^. To our knowledge no such detailed investigation has been previously conducted on spoil heaps generated from hard coal mining.

According to results of previous research the soil enzyme activity and M_w_ index under vegetation should be higher than that in soil without vegetation cover in view of the fact that vegetation can directly or indirectly change soil properties and maintain soil fertility due to the close relationship between plants and microorganisms^78,79^. Our study has partially confirmed this statement since we detected a significantly higher M_w_ index and enzyme activity in plots with vegetation dominated mostly by grasses in comparison to plots that had no vegetation cover (control plots) or to those dominated by forbs. However, Tscherko et al.^80^ postulated that the presence of plants did not stimulate enzyme activity in the pioneer stage of succession, probably due to the severe abiotic stress that plants suffer in these conditions. Plants possess different ecophysiological traits that enable them to utilise soil resources in different ways, furthermore, they have different effects on soil microorganisms^67,81^. In our study the M_w_ index and activity of dehydrogenase as well as alkaline and acid phosphatases was generally higher in plots dominated by grasses (*C. epigejos* and *P. compressa*) compared with plots dominated by other species*.* Han et al.^82^ and Lambers et al.^83^ stated that the significant effect of vegetation on soil biological properties and soil health might be due to the differences in litter input and root exudates. Grasses have a well-developed root system^84^, which is connected with high secretion of root exudates which stimulate the development and activity of communities of microorganisms in the rhizosphere^14,85,86^. In our research, this is shown by higher dehydrogenase activity, which has often been used as an indicator of general soil microbial activity^87,88^. Moreover, in plots dominated by grasses a higher acid phosphatase activity (secreted mainly by plant roots and arbuscular fungi)^89^ and a higher alkaline phosphatase (secreted mainly by bacteria)^90^ were detected compared with plots dominated by forbs. Conversely, Ellhottová et al.^76^ and Stefanowicz et al.^67^ stated that *T. farfara,* a pioneer species on coal mine spoil heaps, significantly increased activity, diversity and biomass of microbial communities in post-mining sites. Our study did not show any difference in the functional diversity of the substrate between vegetated and control plots, while Tscherko et al.^80^ showed that higher functional diversity in the rhizosphere versus the bulk soil in all successional stages indicated greater substrate heterogeneity due to root exudates and root litter.

Plant diversity also had an influence on soil microbial activity and soil quality because each plant species uniquely contributed to the functioning of the below-ground system^18,91^. Rodríguez-Loinaz et al.^53^, in highly diverse native mixed-oak forests, found a negative correlation between the diversity of herbaceous plants and ferns and the activity of acid and alkaline phosphatases in the soil. They explained this fact by suggesting that with sufficient P, plant communities regulated by competitors have lower biodiversity due to competitive exclusion. In the unfavourable conditions which prevail on coal mine spoil heaps (with insufficient available P) we found a positive correlation between the diversity of plants and the activity of soil alkaline phosphatase. Zhang et al.^92^ also found that an increase in the diversity of plants in wetlands caused a higher phosphatase activity in the soil, indicating that a high diversity of plants increases the mineralization rate of organic phosphorus.

### Interactions between above ground vegetation, soil physicochemical properties and enzyme activity of the substrate

As has already been mentioned, during spontaneous succession unfavourable soil conditions (nutrient limitation, moisture) have a greater effect on soil enzyme activity than plant species^80^. Our research has confirmed this statement as we have shown that the soil physicochemical parameters had a bigger impact on the acceleration of biochemical activity in the substrate than biomass and plant species diversity on our hard coal spoil heaps. Chodak and Niklińska^61^ who examined the effect of soil texture and plant species on microbial properties of mine soils also found a predominant influence of soil properties (soil texture) in shaping the enzyme activity. In contrast, the vegetation type and the litter quality appeared to be of higher importance for soil microbial activity than substrate quality on reclaimed heaps after open cast lignite mining^75^. However, in their case, they studied the activity of microorganisms in the substrate of spoil heaps in the later stage of succession (in forest communities). At this stage of the succession, the influence of plants on the enzyme activity of soils is much more significant. The study of Baldrian et al.^1^ showed that there was a general trend towards increasing enzyme activity in the later stages of spontaneous succession on coal mine heaps. Moreover, Allison et al.^93^ found that the activities of soil enzymes increased with site age across carbon and nutrient gradients at the Frantz Josef chronosequence in New Zealand.

## Conclusions

We found that on hard coal spoil heaps the abiotic parameters of the substrate, especially pH and WHC, have a greater effect on the substrate enzyme activity compared to species diversity and biomass associated with the plants. We did not prove our hypothesis about the significant role of vegetation cover that spontaneously developed on hard coal spoil heap on the enzyme activity of the substrate. However, taking into account functional groups of species, grasses, owing to their extensive root system, had a greater influence on the enzyme activity of the substrate than either forbs or a lack of vegetation cover. Moreover, stronger correlations were found between substrate functional diversity, potential biochemical soil fertility index and plant species diversity of plots dominated by grasses than with forbs. Since grasses additionally stabilize the substrate, as well as creating dense and permanent plant cover, they can be recommended for use in the reclamation process of the substrate of hard coal spoil heaps.

Further research is required to gain an understanding of the enzyme activity of a variety of substrates in relation to the changes made by different plant species compositions within the vegetation in order to comprehend the optimal functioning and productivity of this anthropogenic ecosystem. This knowledge will develop an effective tool to assess the environmental conditions of hard coal mining sites and the possibility of enhancing the restoration process based on natural mechanisms in order to ultimately improve the overall biodiversity of the site and its surroundings.
